# Replication Stress in Mammalian Cells and Its Consequences for Mitosis

**DOI:** 10.3390/genes6020267

**Published:** 2015-05-22

**Authors:** Camille Gelot, Indiana Magdalou, Bernard S. Lopez

**Affiliations:** Institut de Cancérologie Gustave Roussy, CNRS UMR 8200 and Université Paris Sud, Equipe labélisée “LIGUE 2014”, 114, Rue Edouard Vaillant, 94805 Villejuif, France; E-Mails: camille.gelot@gustaveroussy.fr (C.G.); indiana.magdalou@gustaveroussy.fr (I.M.)

**Keywords:** replication stress, mitosis, anaphase bridges, single-ended DSB, homologous recombination, centrosome, micronuclei, fragile sites

## Abstract

The faithful transmission of genetic information to daughter cells is central to maintaining genomic stability and relies on the accurate and complete duplication of genetic material during each cell cycle. However, the genome is routinely exposed to endogenous and exogenous stresses that can impede the progression of replication. Such replication stress can be an early cause of cancer or initiate senescence. Replication stress, which primarily occurs during S phase, results in consequences during mitosis, jeopardizing chromosome segregation and, in turn, genomic stability. The traces of replication stress can be detected in the daughter cells during G1 phase. Alterations in mitosis occur in two types: 1) local alterations that correspond to breaks, rearrangements, intertwined DNA molecules or non-separated sister chromatids that are confined to the region of the replication dysfunction; 2) genome-wide chromosome segregation resulting from centrosome amplification (although centrosomes do not contain DNA), which amplifies the local replication stress to the entire genome. Here, we discuss the endogenous causes of replication perturbations, the mechanisms of replication fork restart and the consequences for mitosis, chromosome segregation and genomic stability.

## 1. The Multiple Causes of the Replication Stress

Replication stress has been identified as a very early step for tumorigenesis and senescence [1,2,3,4]. Indeed, cells are exposed daily to endogenous and exogenous stresses. The progression of replication forks (RFs) is routinely challenged by these stresses, leading to stalling, collapse or breakage of replication forks and to genomic instability. Different endogenous sources of stress can affect the progression of replication forks.

### 1.1. Down-Regulation of Limiting Factors of Replication

Faithful DNA replication requires numerous factors, and their limitation can result in the slowing of replication fork progression and ultimately in replication stress. These replication factors include the pool of nucleotides (dNTPs), components of the replication machinery, histones and histone chaperones [5,6]. Moreover, deficiency in active replication origins can also lead to replication stress. Indeed, the replication of origin-poor DNA regions requires long-distance DNA synthesis. The obstacles encountered by replication forks traveling though these regions can lead to the persistence of un-replicated DNA. Notably, this is the case in cells defective in their pre-replication complex assembly [7,8,9,10].

An excess of replication origin firing can also be a source of replication stress through the exhaustion of factors essential for DNA synthesis and for the maintenance of fork integrity, including RPA protein, which protects single-strand DNA (ssDNA) [11,12].

Indeed, the level of RPA becomes limiting when the number of replication origins increases. As a result, new ssDNA stretches cannot be protected by RPA, and therefore, the replication forks become more susceptible to collapse and breakage [13]. The overexpression of RPA can prevent this replication failure [13].

Very subtle perturbations in the level of dNTPs are sufficient to disrupt the rate of replication elongation [14,15]. A decrease in the level of dNTPs has been proposed to be one of the earliest driving forces of tumorigenesis [16,17,18]. It has also been proposed that increasing the number of active replication forks can result in limiting the amount of dNTPs for each progressing replication fork, leading to a genome-wide deceleration in their progression [19].

DNA replication requires also a large amount of histones. Parental nucleosomes are dissociated downstream of the replication fork and are restored on the daughter DNA strands together with newly synthesized histones [20]. Defects in chromatin assembly during DNA replication can disturb the transmission of epigenetic marks, and surprisingly, this can also affect replication dynamics [21]. In *Saccharomyces cerevisiae*, the DNA damage response pathway is required to manage the excess of histones, which could jeopardize genomic stability [22]. In mammals, the supply of neo-synthesized histones regulates the replication elongation rate, possibly through the recycling of the replication factor PCNA [23]. Transient deficiency in neo-synthesized histones induces a decrease in the speed of replication without activating the DNA damage response pathway [23]. Therefore, on one hand, the achievement of the temporal replication program is dependent on a limited quantity of factors. On the other hand, genomes have evolved to have an excess of replication origins to protect cells against replication stress.

Finally, the endogenous production of reactive oxygen species (ROS) as by-products of cellular metabolism can affect the REDOX status of replication factors and alter the initiation or progression of replication [24,25,26,27,28,29,30].

### 1.2. Obstacles to Replication Fork Progression

A wide variety of obstacles can hamper DNA replication by impeding the progression of replication helicases and the capacity of replication polymerases to incorporate new nucleotides. These obstacles include DNA lesions (such as abasic sites, some damaged bases, inter and intra-strand crosslinks), DNA-protein complexes and DNA sequences that can form secondary structures. Some of these obstacles are induced by physiological cellular processes and occur during each S phase. For example, the endogenous production of ROS can generate oxidized bases and abasic sites, and the oxidization of lipids can generate interstrand crosslinks. All of these types of alterations are able to block the progression of polymerases [31].

Another example of a stress generated by a physiological cellular process is the replication of the heterochromatin structure. Heterochromatin (HC) is a transcriptionally repressive environment that is replicated in mid- to late S phase. HC can be distinguished as either facultative HC, which is a cell type-specific chromatin that encodes repressed genes, or constitutive HC, which encompasses the same genomic regions, such as centromeres and telomeres, in all cell types, and consists of repetitive and gene-poor regions. The HC DNA secondary structures, which occur due to repetitive sequences or G-richness, tight DNA-protein complexes and highly compacted chromatin, can hamper polymerase progression.

### 1.3. Interference between Replication and Transcription Machineries/Programs

Transcription represents a major source of endogenous replication stress [6]. Indeed, the replication and transcription machineries share the same DNA template, favoring collisions between the two machineries. Bacterial genomes are generally replicated from a single replication origin, and their genes are co-orientated with the replication fork direction, thus preventing frontal collision between the machineries [32]. In eukaryotes, the situation is complicated by the presence of multiple replication origins. To limit interferences between replication and transcription, these two processes are spatially and temporally separated within the cell nucleus [33]. However, a large number of studies have shown that transcription interferes with DNA replication and can cause genomic instability [6,34].

It has been proposed that replication stress can be induced by DNA/RNA hybrids, which are formed during transcription when the synthesized RNA anneals to the template DNA [35]. Such hybrids are called R-loops and are more likely to occur in GC-rich DNA sequences [36]. R-loops can be resolved by RNAse H and by specific helicases [37,38]. Moreover, the progression of the replication and transcription machineries topologically constrains the DNA, increasing the double helix torsion downstream of the machineries. This leads to the appearance of positive supercoiling [39,40], which can be resolved by topoisomerases 1 and 2 [41]. When the replication and transcription machineries converge towards each other, topological constraints are highly increased and induce the accumulation of positive supercoiling [42], representing a source of replication stress [43,44]. In mammals, very long genes are transcribed over the course of more than one cell cycle [45], and these genes are thus more prone to collisions between the replication and transcription machineries. Most of these genes are replicated during late S phase and correspond to common fragile sites. Interestingly, transcription also disturbs replication at early-replicated sequences. Indeed, a recent genomic study showed the appearance of DNA damage at highly expressed and early-replicated genes following replication stress [46], and these regions have been identified as early replicating fragile sites (ERFS).

## 2. Replication of Particular Regions of the Genome

### 2.1. Under-Replicated DNA and Common Fragile Sites

Common fragile sites (CFSs) are specific loci that preferentially exhibit gaps and breaks upon chromosome condensation during metaphase. These double-strand breaks (DSBs) are visualized by cytogenetic analysis following the exposure of cells to low levels of the polymerase inhibitor aphidicolin (APH), *i.e*., doses that lead to a slowing (two- to ten-fold reduction) in replication fork movement [47,48]. CFSs are found in S. cerevisiae and are conserved in mammalian cells [49,50,51]. CFSs have been highly studied, but the cause of their fragility remains a matter of debate. It was long thought that CFSs contain elements capable of forming secondary structures, such AT-rich sequences that affect the progression of replication forks [52,53,54,55]. However, the suppression of these regions does not avoid breaks in CFSs [48,56,57], and the appearance of CFSs depends on the differentiated cell type [49,58], which argues against a model where the DNA sequence would be responsible for their instability. Genome-wide analysis of replication timing and molecular combing experiments recently allowed the visualization of the replication dynamics of CFSs and showed that CFSs were localized in replication origin-poor regions [59,60,61]. The replication of these regions is based on the capacity to replicate DNA over long distances, and their fragility is correlated with the absence of replication origin firing, which is in agreement with the tissue-specificity of origin firing in those specific regions. Indeed, replication timing is different according to the cell type [62,63,64,65]. Most CFSs correspond to long genes (>300 kb), which might favor the collision of transcription and replication machineries [45] and the tissue-specificity of CFSs. However, it has been demonstrated recently that the transcription of large genes does not systematically dictate CFS fragility [66]. Currently, it is believed that CFSs result from mitotic entry prior to the completion of replication in late-replicating regions, which can correspond to regions that are replication origin-poor [60,67,68,69]. A large number of proteins involved in DNA damage response (DDR), including ATR, polη, BRCA1, RAD51, Claspin, FANC proteins and BLM, are necessary for CFS maintenance [58,70,71,72]. In absence of these proteins and/or upon replicative stress (APH), breaks and anaphase bridges at CFS loci are observed at the following mitosis (see below Section 5.2). However, the mechanism by which checkpoint-proficient cells continue cycling with under-replicated DNA remains unclear.

### 2.2. Telomeric Sequences

The replication of telomeric repeats is ensured by telomerase, but TTAGGG repeats can form G4 DNA structures that block the replication machinery and lead to fork stalling, especially after the addition of aphidicolin [47,73]. Mammalian telomeres are protected from this fragile-site phenotype during replication by the specialized telomeric protein TRF1 [73]. TRF1 has been proposed to inhibit ATR signaling after APH-induced replication stress and to recruit two specialized helicases, BLM and RTEL1, to telomeres to resolve G4 DNA and to avoid the fragile telomere phenotype [73,74]. TRF1-depleted cells are prone to telomeres breakage and chromatid type fusions, that could lead to anaphase bridges formation in mitosis [73,75,76] (see below Section 5.2).

### 2.3. Centromeric Sequences

Centromeres are highly specialized chromosomal structures that hold sister chromatids together during metaphase to ensure their correct alignment on mitotic spindles and kinetochore assembly and, in turn, chromosomal segregation [77]. Centromeric heterochromatin is associated with specific proteins (CENP-A) and consists of repetitive DNA elements, which are tandem arrays of 171 bp, AT-rich alphasatellite monomers that are organized as multimeric, higher-order repeats (HORs) spanning 3–5 Mb [78,79,80]. All of these factors are problematic for the DNA replication machinery, as repeat structures generate topological stress. Indeed, centromeres are known to comprise endogenous sites of replication fork pausing in yeast [81], and they comprise hotspots for chromosomal breakage and rearrangements in mammalian cells [82]. Centromeric DNA might generate higher-order looped structures via recombination between the repetitive elements, which could lead to fully replicated but still intertwined DNA [83,84]. Recent studies have shown that the maintenance of pericentromeric heterochromatin depends of the recruitment of repair proteins during unperturbed S phase (see below Section 3.3).

## 3. Stalled Forks and Replication Restart

As described above, specialized structures or DNA damage can impair the progression of polymerases which leads to an accumulation of long stretches of ssDNA due to the uncoupling of polymerase activity and helicase progression. The ssDNA-binding protein RPA coats the ssDNA that accumulates at stalled forks and in turn recruits the ATR-ATRIP complex. This recruitment activates the DNA replication checkpoint to stabilize the stalled replication fork, arrest cell cycle progression and orchestrate the cellular response for fork restart ensured by homologous recombination (HR). Note that activation of the dormant origins nearby stalled forks overcomes replication slowing and allows to complete replication [85,86].

### 3.1. Regulation of Resection and Homologous Recombination (HR)

**Figure 1:**
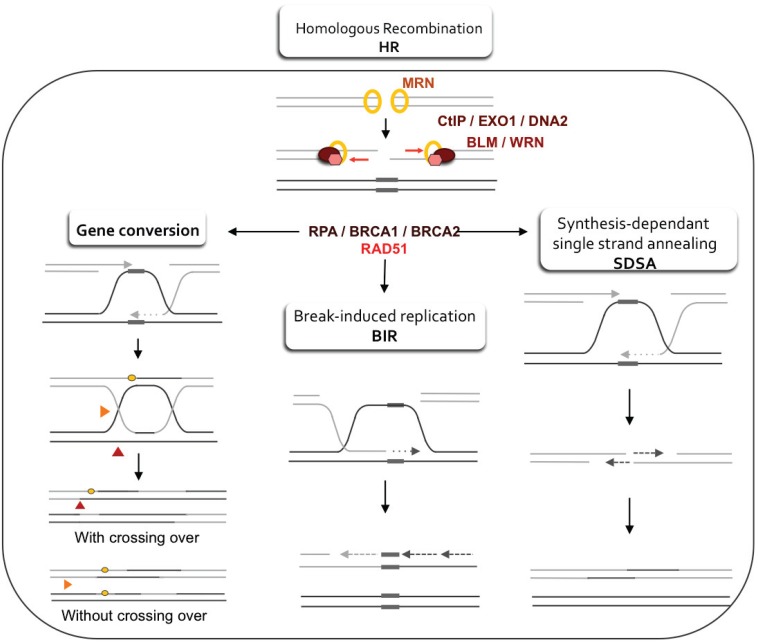
Double-strand break repair models that act via homologous recombination (HR). Left panel: Gene conversion. After resection, the single-stranded 3' tail invades a homologous, intact double-stranded DNA, forming a d-loop (displacement loop). This process tolerates a limited number of imperfect sequence homologies, thus creating heteroduplex intermediates bearing mismatches (yellow circles). The invading 3'-end primes DNA synthesis, which then fills in the gaps. The cruciform junctions (Holliday junctions, HJ) migrate. Resolution (or dissolution) of HJs occurs in two different orientations (orange or red triangles), resulting in gene conversion either with or without crossing over. Middle panel: Break-induced replication (BIR). The initiation is similar to that of the previous models, but the synthesis continues over longer distances on the chromosome arms, even reaching the end of the chromosome. Here, there is neither resolution of the HR nor crossover. Right panel: Synthesis-dependent strand annealing (SDSA). Initiation is similar to that of the previous model, but the invading strand de-hybridizes and re-anneals at the other end of the injured molecule; no HJ is formed.

**Figure 2:**
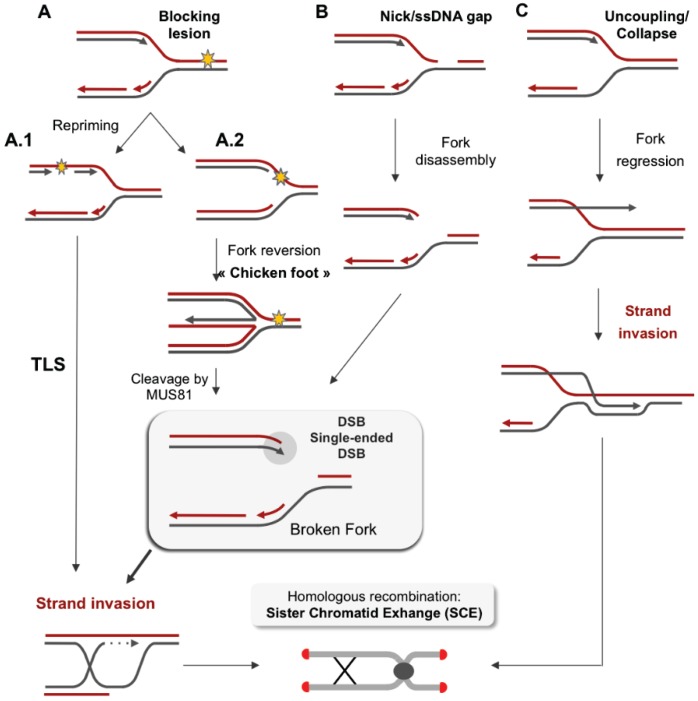
Fork restarts by HR following replication stress. (**A**) *Model of repair of blocking lesions.* (**A.1**) DNA adducts obstruct DNA synthesis by replicative DNA polymerases. Fork progression on a damaged template might involve a repriming event downstream of the damage, which leaves a ssDNA gap behind the moving fork. Rad51 then nucleates on the ssDNA gaps and promotes the recombination with the sister chromatid to seal the gap. Other mechanisms might be involved in the bypass of DNA lesions such as translesion synthesis (TLS). (**A.2**) Model of fork regression at a stalled fork: A slowing down of fork velocity or fork arrest leads to a transient uncoupling of the helicase and polymerases, thus exposing ssDNA at the stalled fork. The fork reversion forms a “chicken foot” structure (*i.e.*, the fork and the nascent strand, which is complementary, being annealed together to form a four-way junction). Cleavage of this structure might involve MUS81 and leads to single-ended DSB formation. (**B**) *Model of broken-fork repair.* A replication fork can be converted into single-ended DSBs following the passage of the fork through a nick or following cleavage by an endonuclease. The single-ended break is then resected and Rad51 nucleates on the exposed ssDNA and promotes recombination with the sister chromatid. The 3' end of the invading strand primes DNA synthesis, and the replisome has been proposed to be rebuilt from the extended d-loop structure. (**C**) *Model of fork restarts at a collapsed fork.* Fork collapse might arise from a stalled fork where the replisome fails to be maintained in a functional state or when the replisome encounters physical obstacles such as tightly DNA bound proteins or RNA/DNA hybrids. Resection of nascent strands might help the fork to regress (*i.e.*, the fork moving backward without the annealing of nascent strands) and thus allow the 3' end of the nascent strand to be extruded. Rad51 nucleates on the exposed ssDNA and promotes recombination with the parental DNA duplex. The replisome could again be rebuilt from the extended d-loop.

HR is an evolutionary process that plays a central role in the equilibrium of genome stability and diversity. HR is involved in the repair of DNA double strand breaks (DSBs) (Figure 1) and in replication fork restart (Figure 2) (see [87] for review).

HR is initiated by a ssDNA 5' to 3' resection that is mediated by MRN (MRE11-RAD50-NBS1)-CtIP, which is followed by the assembly of the RAD51 presynaptic filament, strand invasion of a homologous sequence and the copying of the homologous matrix. Depending on the resolution of the intermediate structure, the final product refers to gene conversion (GC), break-induced replication (BIR) or synthesis-dependent strand annealing (SDSA) (Figure 1).

During replication, the repair of replication stress-induced DSBs is supported by HR [88] and based on the copy of the sister chromatid DNA. HR that occurs between sister chromatids (sister chromatid exchange; SCE) involves identical sequences and therefore does not impact the genetic information. The efficiency of DSB repair by HR is first ensured by resection activation during S/G2. In line with this, silencing CtIP rescues the high level of SCE in Bloom syndrome cells [89]. However, DSB ends are protected from resection by the binding of Ku70/Ku80 and by the 53BP1/RIF1 complex [90,91,92,93,94]. Thus, in S/G2 phase, resection must be activated to counteract Ku70/Ku80- and 53BP1/RIF1-mediated DSE protection. Studies in yeast and mammalian cells have shown that the cyclin-dependent kinase (CDK1)-dependent phosphorylation of CtIP stimulates its activity and results in the activation of resection, the formation of ssDNA and the inhibition of the binding of Ku70/Ku80 to DSBs [95,96,97,98]. In addition, CDK-dependent CtIP phosphorylation enhances its binding with MRN and BRCA1, which promotes the degradation of DNA ends [96,98,99]. The CtIP/BRCA1 complex is crucial for the dissociation of the 53BP1/RIF1 complex [93,99,100,101,102]. Recently, the CDK1/CDK2-dependent phosphorylation of EXO1 was described as being crucial for its recruitment to DSBs and for the activation of resection [103]. Strikingly, BLM plays a double and opposite role: First, it protects against unscheduled resection through its interaction with 53BP1, favoring its loading on the DSB; second, it favors resection during S/G2 *via* its helicase activity and its interaction with TopIIIα, which occurs following a controlled, post-translation modification [89]. Recently, two teams have demonstrated a role for the REV7/MAD2L2 protein in CtIP-dependent inhibition of resection [104,105]. In addition to this small level of resection regulation, the physical proximity of the sister chromatid to the cohesin complex favors sister chromatid exchange and thus maintains genomic stability [106,107,108]. The last known level of HR regulation is in the context of chromatin; DSBs that occur within transcriptionally active chromatin are preferentially repaired by HR [109,110].

### 3.2. Stabilization of the Arrested Replication Fork

During replication stress, the progression of replication forks is limited or blocked. The ATR-CHK1 pathway stabilizes stalled replication forks and prevents their dissociation from the replisome [111,112,113]. A repair-independent role for some of the components of homologous recombination has been recently proposed that involves the protection of nascent DNA at stalled replication forks. Indeed, BRCA1/2 and FANCD2 promote the formation of RAD51 nucleofilaments on ssDNA stretches that are present at stalled replication forks, preventing their resection by MRE11 [19,114,115,116,117,118,119].

A blocked fork can also result in its reversal (Figure 2), as the newly synthetized DNA are complementary. Such structures have been observed in *S. cerevisiae* and in mammals using electron microscopy [120,121,122], and they are thought to protect replication forks from breakage [123]. The reversal of a replication fork can be catalyzed by the helicase SMARCAL1 [124] and is mediated by RAD51 [125].

### 3.3. Restart of the Arrested Replication Fork

Once the source of replication stress is removed, the ATR pathway allows the replication forks to restart [19]. The absence of ATR during replication stress can lead to fork collapse, which is associated with the formation of ssDNA and DSBs at replication forks [126] and to cell death [127]. The local activities of ATR at blocked replication forks remains poorly defined at the molecular level but are the subject of several studies [128,129]. For example, recently, it has been shown that ATR-mediated phosphorylation of FANCI facilitated replication forks restarts [85]. Consistently, in mammals, replication fork restart can be initiated by the loading of HR factors onto the single-strand DNA present at the stalled fork [130,131].

Several different restart pathways could be proposed: (1) fork restart after repriming, *i.e.*, replisome loading after a lesion (Figure 2A.1); (2) restart after a fork reversion, with the newly synthesized DNA strand being homologous to the parental DNA downstream and creating a “chicken foot” structure (Figure 2A.2); and (3) restart using the ssDNA formed after fork regression (Figure 2C) in a process analogous to BIR (Figure 2C). In some cases, single-ended DSBs are formed by either the passage of replication forks through a nick or a ssDNA gap (Figure 2B) or following fork reversion and the cleavage of the reversed forks by structure-specific endonucleases such as MUS81 (Figure 2A.2). Mus81 activity is normally restricted to late G2 or M phase, and the conversion of reversed forks into DSBs is a consequence of premature entry into mitosis [132,133]. HR can then use the sister chromatid to prime DNA synthesis, allowing for the resumption of replication (Figure 2).

In agreement with the fact that centromeric repeats challenge the progression of replication, BRCA1 has been shown to localize at or near pericentromeric HCs during S phase, which suggests a role of the HC region during DNA replication in mammalian cells [134]. RIF1 has also been shown to localize with RPA at pericentromeric regions, particularly following replication stress via an ATR dependent pathway and to promote homology-directed repair. These data suggest a role for RIF1 in the repair of stalled forks via the facilitation of HR [135]. Moreover, in *S. pombe*, Rad51 is recruited to centromeres during S phase. It has been suggested that this recruitment enables the gene conversion-mediated repair of repeated centromeric sequences and preserves cells from isochromosome formation [136]. Note that similar to telomeres, centromeres are highly repetitive sequences and recombinogenic [136,137].

Thus, HR is an essential mechanism for the protection, recovery and restart of replication forks. This important role during DNA replication is underscored by the phenotypes of HR-deficient cells: Slow replication fork speed [15,138] and mitotic defects. However, while it is crucial for maintaining genetic stability, HR can prime error-prone DNA synthesis and favor rearrangements as previously discussed [139].

### 3.4. Error-Prone Replication Forks Restart

**Figure 3:**
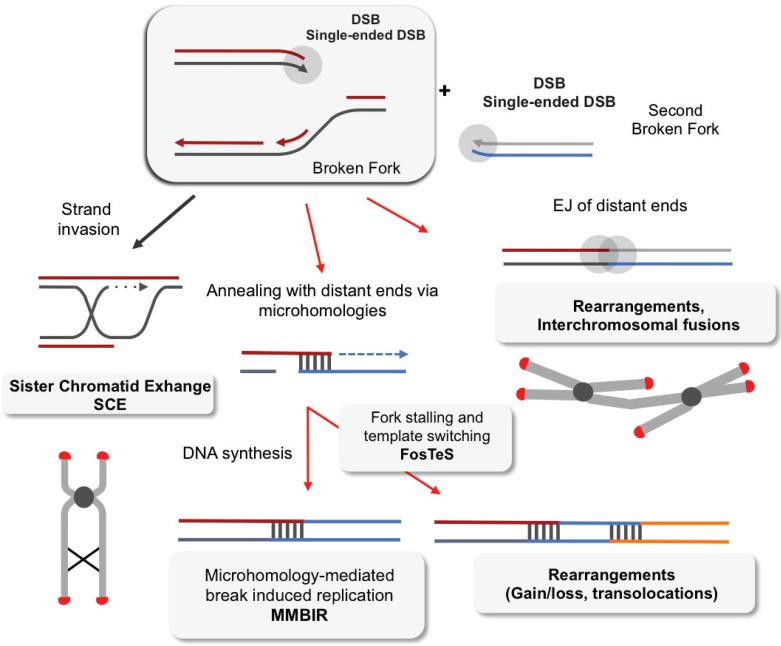
Joining of single-ended double strand breaks (DSBs) could lead to rearrangements. (**Left panel**): A single-ended DSB generated by replication stress is normally repaired by SCE in a conservative way. Rearrangements occur when a single-ended DSB is joined to another single-ended DSB, which is likely to be distal. (**Middle panel**): The annealing of few nucleotides at the extremity of the single ended DSB with another broken fork activates the MMBIR (microhomology-mediated break-induced replication) mechanism. MMBIR coupled to several switches in fork templates leads to complex rearrangements and has been proposed to be a mechanism that originates chromotripsis. (**Right panel**): The end-joining (EJ) by C-NHEJ or A-EJ of the single ended DSB with another single-ended DSB lead to dicentric chromosome formation.

In some cancer cells, complex genomic rearrangement has been observed and named chromothripsis. Chromothripsis (chromo for chromosome and thripsis for breaking into small pieces) is characterized by the shattering of one or more chromosomal segments followed by the chaotic reassembly of the fragments; both events occur during one unique cellular event [140,141,142]. Some chromothripsis events might be the result of chromosome shattering followed by end-joining (Figure 3) of the DSBs via non-homologous end-joining (NHEJ) or alternative end-joining (A-EJ) [139,141]. However, gain of DNA, such as sequences duplication, observed at chromothripsis loci obliged to also consider some DNA synthesis steps.

MBIR (microhomology-mediated break-induced replication) [143,144] associated with a specific mechanism linked to a block in replication, FoSTeS (fork stalling and template switching) [145] can produce complex rearrangements (Figure 3). These processes begin with the conversion of a DSB in a 3' ssDNA stretch. This free 3' DNA end can then anneal using a region of micro-homology (a few nucleotides in length) on a region of ssDNA that is exposed on an adjacent replication fork, which allows replication to resume (template switching) (Figure 3).

Such replication forks can undergo several rounds of template switching, generating complex rearrangements with deletions, amplifications and non-reciprocal translocations. The use of this low-fidelity repair process to manage the significant number of DSBs that are generated during chromothripsis could be the result of reliable repair processes and DDRs becoming overwhelmed.

## 4. Impact of Single-Ended DSBs Formed by Replication Stress on Chromosome Instability

### 4.1. DSB Repair

**Figure 4:**
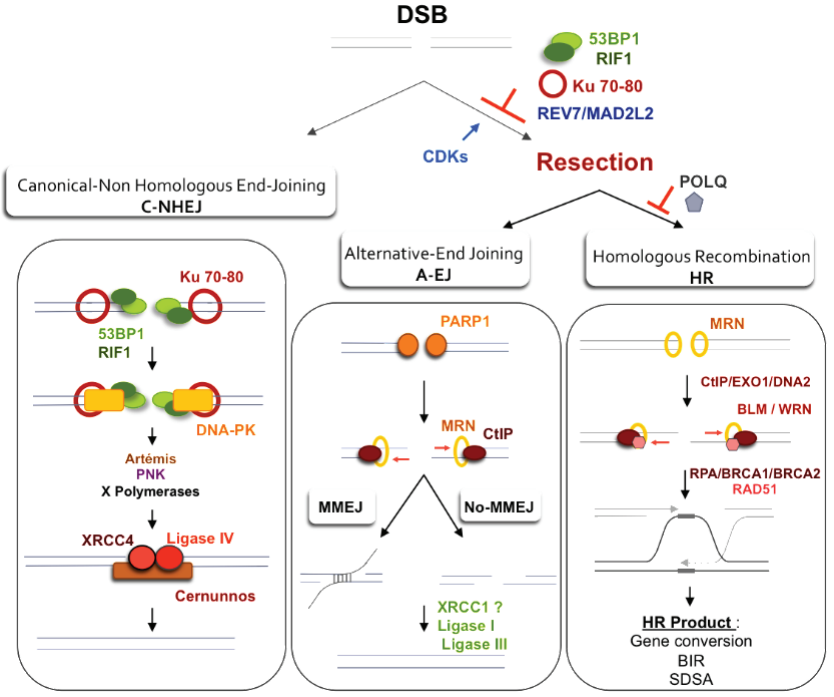
DSB repair pathway models. (**Left panel**): *Canonical C-NHEJ*. The heterodimer Ku80-Ku70 binds to DNA ends, which then recruits DNA-PKcs. In subsequent steps, several proteins including Artemis, polynucleotide kinase (PNK), and members of the polymerase X family process the DNA ends. In the last step, ligase IV associated with its co-factors Xrcc4 and Cernunos/XLF joins the ends (for review about cNHEJ and A-EJ actors see [148,151]). (**Right Panel**): *Resection as a common initiation step for HR and A-EJ at DSB.* 53BP1, RIF1 and Ku70-80 heterodimer protect DSB ends from resection and HR and A-EJ actions. The CDK1/2-dependent phosphorylation of CtIP and EXO1 favors the initiation of resection and extension, respectively [95,103,152]. Recently, REV7/MAD2L2 was described as an inhibitor of resection and HR, although its role in A-EJ inhibition was not directly studied and remains hypothetical [104,105]. A short ssDNA resection allows for A-EJ but not homologous recombination, while a long ssDNA resection allows for both A-EJ and HR; however, HR requires the presence of homologous sequences. Recently, POLQ polymerase was shown to inhibit HR and to promote A-EJ at DSBs [153,154]. A-EJ results in repair that is error-prone and is associated with deletions at the repair junctions with frequent use of microhomologies that are distant from the DSB. *Alternative-EJ:* Parp1 plays a role in the initiation process, and it has been proposed that a single-strand DNA resection reveals complementary microhomologies (two to four nucleotides or more in length) that can anneal, with gap-filling completing the end-joining. A-EJ is always associated with deletions at the junctions and can involve microhomologies (MMEJ or microhomologies-mediated EJ) that are distant from the DSB. Subsequently, Xrcc1 and ligase III (which can be substituted by ligase I) complete the A-EJ process. *Homologous recombination:* The first step, which is the initiation of resection, involves the removal of ~50–100 bases of DNA from the 5' end by the MRN complex (Mre11-Rad50-Nbs1) in conjunction with CtIP. The second step, resection extension, is carried out by two alternate pathways involving either the 5' to 3' exonuclease EXO1 or the helicase-topoisomerase complex BLM-TOPIIIα-RMI1-2 in concert with the nuclease CtIP/DNA2. WRN helicase has also been shown to act with CtIP and to stimulate resection in human cells [155].

Replication stress can generate DNA double-strand breaks through several mechanisms: Replication forks reaching a ssDNA nick or gap or the resolution by the structure-specific nuclease MUS81 of the intermediate (chicken foot) that is generated by the reversion of the arrested replication fork [146,147] (Figure 2 and Figure 3). DSB are highly toxic lesions that can generate genomic rearrangements and can challenge cell fate. DSBs can be repaired by canonical non-homologous end joining (C-NHEJ), a Ku70/Ku80 and XRCC4-DNA Ligase 4 (Lig4)-dependent mechanism that joins the DNA double-strand ends (DSE) without requiring sequence homology (Figure 4) or by HR (Figure 1 and Figure 4). More recently, another mechanism of DSB repair has been described: The alternative end-joining (A-EJ), which is initiated by MRN/CtIP resection but does not require sequence homology to seal the broken ends (Figure 4). A-EJ is a highly mutagenic process leading to deletions that are frequently, but not always, associated with micro-homologies at the repair junction (for review see [148]). A-EJ is highly repressed by C-NHEJ and by several proteins such as 53BP1, RIF1, BLM, PTIP and the recently described REV7/MAD2L2 [89,93,100,101,104,105,149], which protect the ends against resection. End-joining (EJ) and HR are both critical for stability and cell survival but can also drive genomic instability. Both processes also manage repair of programmed DSBs that are generated during physiological mechanisms aimed at generating genetic diversity such as meiosis, V(D)J recombination and class-switch recombination (CSR) (for reviews see; [139,148,150]). Therefore, the choice between HR and EJ is essential for efficient and accurate repair, and several types of regulation have been described: (1) resection activation/repression; (2) physical proximity of the sister chromatid; and (3) active gene and chromatin conformation.

### 4.2. EJ of Distant DBS Ends Leads to Radial Structure Formation and Dicentric Chromosomes

A DSB generated by replication stress exhibits only one single DSE. The rejoining of this DSE with another, distal DSE will inexorably result in genomic rearrangements such as dicentric chromosomes, radial structures and/or translocations (Figure 3). Indeed, it has been shown that NHEJ can process DSBs generated by replication stress [88,156,157]. Moreover, radial structures, which can occur spontaneously in BRCA1- or Fanconi-deficient cells (thus arising from endogenous replication stress), are suppressed upon the inhibition of Ku70, Ku80, DNA-PKcs or LIGIV, demonstrating the role of NHEJ in the formation of such chromosomal aberrations [93,158,159]. Consistently, fusions of deprotected telomeres, which lead to the formation of dicentric chromosomes, depending on EJ proteins such as Ku70 and DNA ligase 4 [160,161,162]. Therefore, the restriction of EJ for distant DSEs should protect against genomic rearrangement. The restriction of DSE mobility could be one factor that restrains the synapsis of distant DSEs and subsequent EJ. Studies in yeast and mammalian cells have indicated that chromatin domains containing IR-induced DSBs can be mobile [163,164,165]. Several teams have investigated this DNA mobility in living mammalian cells by measuring the dynamics of a single DSB at a defined genomic site or the mean squared displacement (MSD) of tagged repair proteins. It has been found that DNA repair proteins such as Ku70 or ATM are directly involved in the restriction of DNA end mobility [166,167,168,169]. Moreover, a study of translocation event formation, *i.e.,* the joining of distant DSEs, revealed a crucial role for MRE11 in the pairing of distal DSEs [167].

## 5. Consequences of Replication Stress on Mitosis

Replication stress, which primarily occurs during S phase, can have deleterious consequences on the subsequent phases of the cell cycle such as mitosis, ultimately affecting faithful chromosome segregation. Mitotic alterations can be classified in two categories: (1) local alterations such as breaks and anaphase bridges and (2) genome-wide alterations resulting from the generation of supernumerary centrosomes and leading to multipolar uneven segregation. Although the centrosome does not contain DNA, extra mitotic centrosomes can amplify local replication stress to genome-wide instability. Indeed, multipolar centrosomes are associated with mitotic delay and anaphase bridges. Even very low levels of replication stress have been shown to induce mitotic defects such as anaphase bridges, extra mitotic centrosomes and multipolar mitosis, leading to uneven chromosome segregation and aneuploidy [15,70].

### 5.1. A Threshold of Stress for S and G2/M Arrests

Damage created during S or G2 phase normally leads to a checkpoint arrest through ATR and ATM activation, and this process is essential for repair and possible re-entry into mitosis. In checkpoint-proficient cells, ATR pathway sensors and mediators are loaded to DNA in cases of moderate replication stress, *i.e.*, a two- to ten-fold reduction of fork speed (0.038 to 0.6 µM aphidicolin). However, the phosphorylation of p53, CHK1, ATM, CHK2 or RPA2 remains undetectable, reflecting the absence of activation of DDR. Therefore such a stress fails to block mitotic entry and leads chromosome breaks at under-replicated region, after metaphase condensation [72]. Thus, moderate stress is not sufficient to induce the DDR response and cell cycle arrest. Note that ATR could play a role in the delay of mitotic entry with or without replicative stress.

Moreover, it has been recently shown that another checkpoint that is dependent on p53/p21 leads to cell senescence following damage by inducing nuclear translocation and the degradation of Cyclin B1 protein, which is essential for mitosis entry [170,171]. Note that following damage (etoposide or IR), CyclinB1 is accumulated in the nucleus, but its degradation is only initiated once a certain “threshold” of damage is reached, leading to an irreversible cell cycle exit [171].

### 5.2. Anaphases Bridges

**Figure 5:**
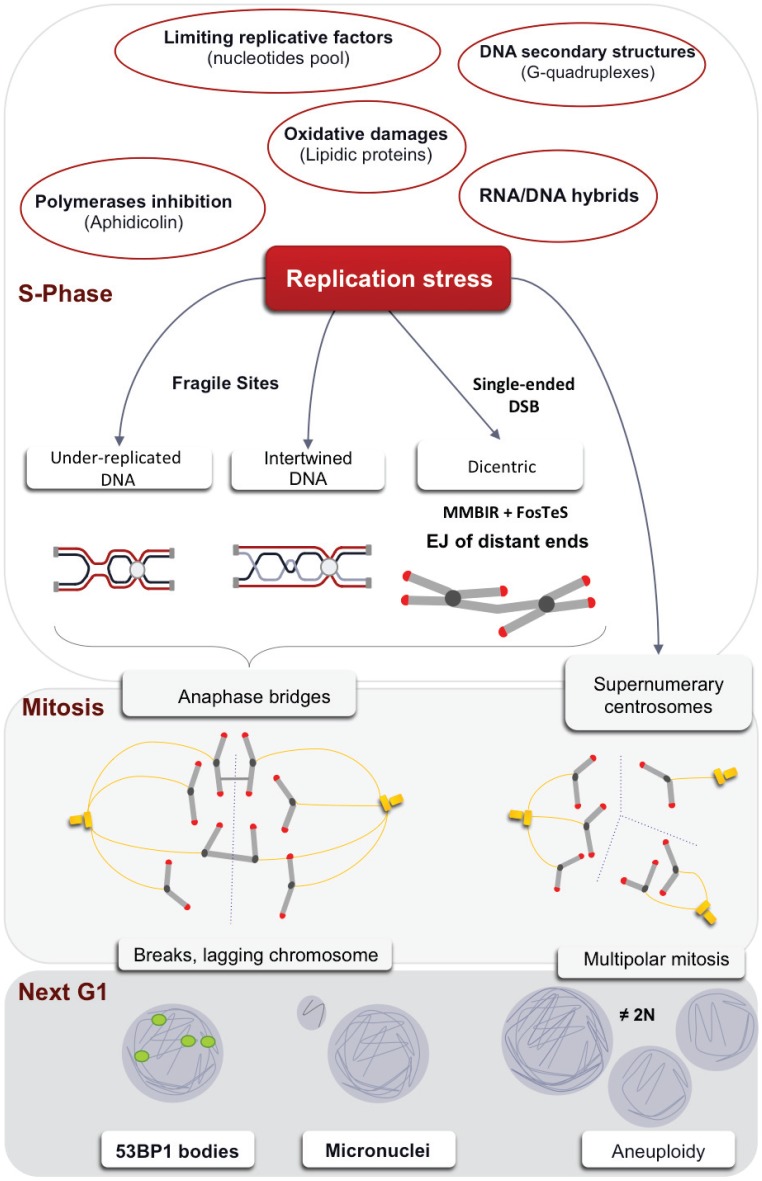
Replicative stress and its consequences in mitosis. Replication stress from either endogenous or exogenous causes (red circles) leaves chromosomal segment unreplicated or interwinded leading to anaphase bridges formation. Single-ended DSB could lead to dicentric chromosome formation and thus, also, to anaphase bridge formation. Non-detected damages upon low replicative stress could be grouped in one detectable entity in G1: 53BP1 bodies and/or micronuclei. Replication stress also favors mitotic extra centrosomes and multipolar mitosis, thus amplifying mitotic catastrophes and genome instability to the whole genome.

Non-fully replicated, intertwined DNA regions can reach mitosis and thus transmit replication stress from S phase to mitosis. Such structures can impair the disjunction of sister chromatids. Failure to correctly and completely untangle chromatid sisters can lead to the formation of anaphase bridges (Figure 5).

There are two types of anaphase bridges. The first type can be labeled by intercalating agents such as DAPI and either occur spontaneously in HR-defective cells or can be induced by replication stress, even at very low doses [15]. The second type of anaphase bridge, called an ultra-fine bridge (UFB), cannot be detected by DAPI but can be detected by immunostaining for proteins such as RPA, PICH (PLK1-interacting checkpoint helicase), BLM, or proteins of the FANC complex [70,172,173,174]. Breaks in under-replicated CFS regions have been shown to result from the actions of MUS81-EME1, ERCC1 and SLX4 during metaphase, which avoids the formation of anaphase bridges during chromosomal segregation [175,176,177].

CFS, telomere (T-UFB) or centromere (C-UFB) sequences can be detected in ultra-fine bridges. These regions are proposed to correspond with under-replicated and/or unresolved repair intermediates [172,176]. It has been shown that BLM and other associated proteins (TopoIIIα, RMI1 and RMI2) play a crucial role in the resolution of centromeric and non-centromeric anaphase bridges. The complex can branch-migrate and decatenate entangled DNA, preventing defective chromosome segregation under conditions of replication stress [70,172,178]. Moreover, the resolution of non-centromeric anaphase bridges requires the collaboration of BLM with Fanconi anemia (FANC) proteins [70]. At telomeres, the RecQ helicase WRN (Werner) and BLM act synergistically to process late-replicating intermediates [179]. Constituently, anaphase bridges are generated following replication stress and occur spontaneously in BLM-, FANC- and WRN-deficient cells [70,174,179] and also in HR-deficient cells [180,181,182]. The appearance of anaphase bridges can also occur in response to endogenous or very low level replication stresses that escape cell surveillance pathways [15,72]. Note that interchromosomal fusions that lead to dicentric chromosomes can also form anaphase bridges [183,184].

The disjunction of chromosomes during anaphase creates an increasing mechanical tension on intertwined chromosomes due to unresolved repair or replication intermediates and sister chromatids that are not disjointed, and this tension can induce the mitotic checkpoint [185]. During a prolonged mitotic arrest, this tension can induce chromosomal breaks, thus leading to aberrant chromosome segregation and genetic instability. In mammalian cells, even very low levels of replication stress resulted in prolonged metaphase arrest followed by the formation of anaphase bridges and multipolar chromosome segregation [15]. Note that in the absence of breaks, the non-disjunction of sister chromatids during anaphase leads to nucleoplasmic bridges (observable during cytokinesis) and possibly to aneuploidy, with both chromatids being pulled toward the same mitotic spindle pole [186].

### 5.3. Centrosome Defects

Although centrosomes do not contain DNA, HR-deficient cells show a spontaneous decrease in replication speed [15,138] and an increase of the number of mitotic extra centrosomes [187,188,189,190]. It is noteworthy that these extra centrosomes are systematically associated with chromosome bridges, prolonged metaphase arrest and multipolar chromosomal segregation [15] (Figure 5). Importantly, extra mitotic centrosomes result from the endogenous replication stress that occurs in HR-defective cells. Indeed, low levels of replication stress mimics the replication fork speed of HR-defective cells and generates the same mitotic defects. Reciprocally, rescuing the slow replication speed of HR-defective cells by supplying deoxynucleoside also rescues extra mitotic centrosomes and mitotic defects [15].

Although DNA replication and centrosome duplication take place at the same time, they are two mechanisms that can be uncoupled [191,192,193]. It has been shown that the inhibition of the ATR-CHK1 pathway reduces the frequency of extra mitotic centrosomes in HR-deficient cells [194].

Similar to endogenous replication stress, supernumerary centrosomes have been observed in precancerous lesions [195,196,197,198,199].

## 6. Transmission of the Stress Signal to the Daughter Cell

Replication stress from either endogenous or exogenous causes (red circles) leaves chromosomal segments unreplicated or intertwined, which leads to the formation of anaphase bridges. Single-ended DSBs could lead to dicentric chromosome formation and thus to the formation of anaphase bridges. Undetected damage following low replicative stress could be grouped in one detectable entity in G1: 53BP1 bodies and/or micronuclei. Replication stress also favors extra mitotic centrosomes and multipolar mitosis, thus amplifying mitotic catastrophes and genomic instability.

### 6.1. Micronuclei

First observed in erythrocytes by William Howell and Justin Jolly [200,201], Howell-Jolly bodies or micronuclei (MN) are extranuclear bodies that are observable in daughter cells following cytokinesis [70,202,203,204,205]. MN are the consequence of lagging chromosomes, acentric chromosomes or chromatid fragments that have been embedded into their own nuclear envelope [206,207,208] (Figure 5). Importantly, in normal cells, replication stress induces MN formation [209]. Consequently, cells lacking proteins that are involved in the resolution of anaphase bridges, such as the FANC pathway and BLM, show a higher rate of MN formation [70]. Moreover, it has been shown that MN can persist in cells over several generations and undergo asynchronous replication that leads to extensive fragmentation of MN DNA [140]. It has been proposed that the re-integration of DNA pieces that are contained within MNs into the cell genome could also help to explain the chromothripsis phenomenon [140,141,142]. Note that in p53-proficient cells, MN-generated aneuploidy leads to cell cycle arrest and apoptosis [210,211].

### 6.2. 53BP1 Bodies

Following replication stress, chromosome damage can bypass mitosis and can be transmitted to daughter cells. During the next G1 phase, this damage is associated with the formation of nuclear bodies, commonly called 53BP1 bodies, containing proteins of the DNA damage response including the 53BP1, γH2AX, MDC1 and OPT domains (Oct-1, PFT, transcription) [212,213]. Notably, these 53BP1 bodies contain damaged CFSs, and the frequency of these 53BP1-associated CFS bodies increases following replication stress that is coupled to a depletion of ERCC1 and/or MUS81 [175]. These data suggest that 53BP1 bodies contain CFS breaks that result from replication intermediates that are not processed prior to anaphase. Consistent with their roles in CFS maintenance and/or the resolution of anaphase bridges, the inhibition of BLM, FANCD2 or polη also increases the frequency of 53BP1 bodies in daughter cells [71,175,213]. Therefore, it has been proposed that 53BP1 bodies could gather non-repaired mitotic DSBs to allow for their repair during G1 phase by C-NHEJ or through recombination during the next S phase, which would restore cell integrity [213]. Indeed, DSB repair is abolished in mitotic cells, resulting in the transmission of DSBs to the following G1 phase. During mitosis, PLK1 and CDK1 phosphorylate 53BP1 and RNF8, which impairs the recruitment of DDR proteins to DSBs and therefore inhibits DSB repair prior to the exit from mitosis [214,215,216].

## 7. Conclusions

Replication stress has been shown to occur during early stages of tumorigenesis or senescence [1,2,3,4]. More recently, genetic instability in colorectal cancer cell lines has revealed a direct link between replication stress and cancer chromosomal instability (CIN) [3]. Strikingly, cells bearing incompletely replicated regions, DNA damage or DNA intermediates can reach mitosis. Importantly, a threshold of DNA damage should be reached to efficiently activate cell cycle checkpoints; consequently, low or endogenous stress levels can remain undetected, which allows cells with DNA damage to reach mitosis.

Cells reaching mitosis with damaged DNA or incompletely replicated regions can form anaphase bridges and chromosomal breaks, resulting in CSF expression and in the formation of 53BP1 bodies and MN in the daughter cells during G1 phase.

In addition, although centrosomes do not contain DNA, replication stress generates supernumerary centrosomes during mitosis in association with anaphase bridges. Such supernumerary centrosomes lead to multipolar, uneven chromosomal segregation and to aneuploidy and genomic instability in daughter cells. Thus, the formation of extra centrosomes amplifies the local stress to the whole genome. Remarkably, as with replication stress, extra centrosomes have been observed in precancerous lesions and tissues adjacent to tumors, suggesting a role in tumorigenesis [195,196,197,198,199].

In 1914, Theodor Boveri observed supernumerary centrosomes and abnormal mitosis in sea urchin embryos. He proposed that supernumerary centrosomes can lead to aberrant chromosomal segregations and aneuploidy and proposed the theory of the clonal origin of tumors [217,218]. The mechanisms linking S-phase progression and centrosome duplication are poorly understood, and the elucidation of how chronic and/or low replication stress promotes centrosomal amplification in mitosis constitutes exciting challenges for future studies.

## Abbreviations

53BP1: P53 Binding Protein 1
A-EJ: Alternative End-Joining
APH: Aphidicolin
BIR: Break-induced replication
C-NHEJ: Canonical Non Homologous End-Joining
CENP: Centromer protein
CDK: Cyclin Dependant kinase
CFSs: Common fragile sites
CIN: Chromosomal instability
DDR: DNA damage response
D-loop: Displacement loop
dNTPs: Deoxynucleotides
DSBs: Double-strand breaks
DSE: Double-strand end
EJ: End-Joining
ERFS: Early replicating fragile sites
FANC: Fanconi
FoSTeS: Fork Stalling and Template Switching
GC: Gene conversion
HC: Heterochromatin
HP: Heterochromatin Protein
HR: Homologous Recombination
HORs: Higher-Order Repeats
HJ: Holliday junctions
IR: Ionizing Radiation
MH: Microhomology
MMBIR: Microhomology-Mediated Break Induced Replication
MMEJ: Microhomology-Mediated End Joining
MN: MicroNuclei
MRN: MRE11/RAD50/NBS1 complex
OPT: Oct-1, PTF, transcription
ROS: Reactive Oxygen Species
SCE: Sister chromatid exchange
ssDNA: Single-Strand DNA
SDSA: Synthesis-Dependent Strand Annealing
T-SCE: Telomere Sister Chromatid Exchange
TLS: Translesion Synthesis
UFB: Ultra-Fine Bridge

## References

[1] Gorgoulis V.G., Vassiliou L.F., Karakaidos P., Zacharatos P., Kotsinas A., Liloglou T., Venere M., Ditullio R.A., Kastrinakis N.G., Levy B. (2005). Activation of the DNA damage checkpoint and genomic instability in human precancerous lesions. Nature.

[2] Bartkova J., Horejsí Z., Koed K., Krämer A., Tort F., Zieger K., Guldberg P., Sehested M., Nesland J.M., Lukas C. (2005). DNA damage response as a candidate anti-cancer barrier in early human tumorigenesis. Nature.

[3] Burrell R.A., McClelland S.E., Endesfelder D., Groth P., Weller M.-C., Shaikh N., Domingo E., Kanu N., Dewhurst S.M., Gronroos E. (2013). Replication stress links structural and numerical cancer chromosomal instability. Nature.

[4] Flach J., Bakker S.T., Mohrin M., Conroy P.C., Pietras E.M., Reynaud D., Alvarez S., Diolaiti M.E., Ugarte F., Forsberg E.C. (2014). Replication stress is a potent driver of functional decline in ageing haematopoietic stem cells. Nature.

[5] Anglana M., Apiou F., Bensimon A., Debatisse M. (2003). Dynamics of DNA replication in mammalian somatic cells: Nucleotide pool modulates origin choice and interorigin spacing. Cell.

[6] Aguilera A., García-Muse T. (2013). Causes of genome instability. Annu. Rev. Genet..

[7] Bruschi C.V., McMillan J.N., Coglievina M., Esposito M.S. (1995). The genomic instability of yeast cdc6-1/cdc6-1 mutants involves chromosome structure and recombination. Mol. Gen. Genet..

[8] Loupart M.L., Krause S.A., Heck M.S. (2000). Aberrant replication timing induces defective chromosome condensation in Drosophila ORC2 mutants. Curr. Biol..

[9] Tanaka S., Diffley J.F.X. (2002). Deregulated G1-cyclin expression induces genomic instability by preventing efficient pre-RC formation. Genes Dev..

[10] Shima N., Alcaraz A., Liachko I., Buske T.R., Andrews C.A., Munroe R.J., Hartford S.A., Tye B.K., Schimenti J.C. (2007). A viable allele of Mcm4 causes chromosome instability and mammary adenocarcinomas in mice. Nat. Genet..

[11] Wold M.S. (1997). Replication protein A: A heterotrimeric, single-stranded DNA-binding protein required for eukaryotic DNA metabolism. Annu. Rev. Biochem..

[12] Fanning E., Klimovich V., Nager A.R. (2006). A dynamic model for replication protein A (RPA) function in DNA processing pathways. Nucleic Acids Res..

[13] Toledo L.I.I., Altmeyer M., Rask M.-B., Lukas C., Larsen D.H.H., Povlsen L.K.K., Bekker-Jensen S., Mailand N., Bartek J., Lukas J. (2013). ATR prohibits replication catastrophe by preventing global exhaustion of RPA. Cell.

[14] Poli J., Tsaponina O., Crabbé L., Keszthelyi A., Pantesco V., Chabes A., Lengronne A., Pasero P. (2012). dNTP pools determine fork progression and origin usage under replication stress. EMBO J..

[15] Wilhelm T., Magdalou I., Barascu A., Técher H., Debatisse M., Lopez B.S. (2014). Spontaneous slow replication fork progression elicits mitosis alterations in homologous recombination-deficient mammalian cells. Proc. Natl. Acad. Sci. USA.

[16] Gad H., Koolmeister T., Jemth A.-S., Eshtad S., Jacques S.A., Ström C.E., Svensson L.M., Schultz N., Lundbäck T., Einarsdottir B.O. (2014). MTH1 inhibition eradicates cancer by preventing sanitation of the dNTP pool. Nature.

[17] Bester A.C., Roniger M., Oren Y.S., Im M.M., Sarni D., Chaoat M., Bensimon A., Zamir G., Shewach D.S., Kerem B. (2011). Nucleotide deficiency promotes genomic instability in early stages of cancer development. Cell.

[18] Saldivar J.C., Miuma S., Bene J., Hosseini S.A., Shibata H., Sun J., Wheeler L.J., Mathews C.K., Huebner K. (2012). Initiation of genome instability and preneoplastic processes through loss of Fhit expression. PLoS Genet..

[19] Petermann E., Orta M.L., Issaeva N., Schultz N., Helleday T. (2010). Hydroxyurea-stalled replication forks become progressively inactivated and require two different RAD51-mediated pathways for restart and repair. Mol. Cell.

[20] Groth A., Rocha W., Verreault A., Almouzni G. (2007). Chromatin challenges during DNA replication and repair. Cell.

[21] Clemente-Ruiz M., González-Prieto R., Prado F. (2011). Histone H3K56 acetylation, CAF1, and Rtt106 coordinate nucleosome assembly and stability of advancing replication forks. PLoS Genet..

[22] Gunjan A., Verreault A. (2003). A Rad53 kinase-dependent surveillance mechanism that regulates histone protein levels in *S. cerevisiae*. Cell.

[23] Mejlvang J., Feng Y., Alabert C., Neelsen K.J., Jasencakova Z., Zhao X., Lees M., Sandelin A., Pasero P., Lopes M., Groth A. (2014). New histone supply regulates replication fork speed and PCNA unloading. J. Cell Biol..

[24] Choi T.-Y., Park S.Y., Kang H.-S., Cheong J.-H., Kim H.-D., Lee B.L., Hirose F., Yamaguchi M., Yoo M.-A. (2004). Redox regulation of DNA binding activity of DREF (DNA replication-related element binding factor) in Drosophila. Biochem. J..

[25] Girard P.-M., Pozzebon M., Delacôte F., Douki T., Smirnova V., Sage E. (2008). Inhibition of S-phase progression triggered by UVA-induced ROS does not require a functional DNA damage checkpoint response in mammalian cells. DNA Repair.

[26] Montaner B., O’Donovan P., Reelfs O., Perrett C.M., Zhang X., Xu Y.-Z., Ren X., Macpherson P., Frith D., Karran P. (2007). Reactive oxygen-mediated damage to a human DNA replication and repair protein. EMBO Rep..

[27] Onn I., Milman-Shtepel N., Shlomai J. (2004). Redox potential regulates binding of universal minicircle sequence binding protein at the kinetoplast DNA replication origin. Eukaryot. Cell.

[28] Sanders C.M., Sizov D., Seavers P.R., Ortiz-Lombardía M., Antson A.A. (2007). Transcription activator structure reveals redox control of a replication initiation reaction. Nucleic Acids Res..

[29] Wang M., You J.S., Lee S.H. (2001). Role of zinc-finger motif in redox regulation of human replication protein A. Antioxid. Redox Signal..

[30] Weiner B.E., Huang H., Dattilo B.M., Nilges M.J., Fanning E., Chazin W.J. (2007). An iron-sulfur cluster in the C-terminal domain of the p58 subunit of human DNA primase. J. Biol. Chem..

[31] Wallace S.S. (2002). Biological consequences of free radical-damaged DNA bases. Free Radic. Biol. Med..

[32] Lin Y.-L., Pasero P. (2012). Interference between DNA replication and transcription as a cause of genomic instability. Curr. Genomics.

[33] Wei X. (1998). Segregation of transcription and replication sites into higher order domains. Science.

[34] Zeman M.K., Cimprich K.A. (2014). Causes and consequences of replication stress. Nat. Cell Biol..

[35] Huertas P., Aguilera A. (2003). Cotranscriptionally formed DNA:RNA hybrids mediate transcription elongation impairment and transcription-associated recombination. Mol. Cell.

[36] Ginno P.A., Lim Y.W., Lott P.L., Korf I., Chédin F. (2013). GC skew at the 5' and 3' ends of human genes links R-loop formation to epigenetic regulation and transcription termination. Genome Res..

[37] Wu H., Lima W.F., Crooke S.T. (1999). Properties of cloned and expressed human RNase H1. J. Biol. Chem..

[38] Alzu A., Bermejo R., Begnis M., Lucca C., Piccini D., Carotenuto W., Saponaro M., Brambati A., Cocito A., Foiani M. (2012). Senataxin associates with replication forks to protect fork integrity across RNA-polymerase-II-transcribed genes. Cell.

[39] Baker T.A., Sekimizu K., Funnell B.E., Kornberg A. (1986). Extensive unwinding of the plasmid template during staged enzymatic initiation of DNA replication from the origin of the *Escherichia coli* chromosome. Cell.

[40] Kouzine F., Gupta A., Baranello L., Wojtowicz D., Ben-Aissa K., Liu J., Przytycka T.M., Levens D. (2013). Transcription-dependent dynamic supercoiling is a short-range genomic force. Nat. Struct. Mol. Biol..

[41] Bermejo R., Doksani Y., Capra T., Katou Y.-M., Tanaka H., Shirahige K., Foiani M. (2007). Top1- and Top2-mediated topological transitions at replication forks ensure fork progression and stability and prevent DNA damage checkpoint activation. Genes Dev..

[42] Olavarrieta L., Hernández P., Krimer D.B., Schvartzman J.B. (2002). DNA knotting caused by head-on collision of transcription and replication. J. Mol. Biol..

[43] Tuduri S., Crabbé L., Conti C., Tourrière H., Holtgreve-Grez H., Jauch A., Pantesco V., de Vos J., Thomas A., Theillet C. (2009). Topoisomerase I suppresses genomic instability by preventing interference between replication and transcription. Nat. Cell Biol..

[44] Bermejo R., Capra T., Gonzalez-Huici V., Fachinetti D., Cocito A., Natoli G., Katou Y., Mori H., Kurokawa K., Shirahige K. (2009). Genome-organizing factors Top2 and Hmo1 prevent chromosome fragility at sites of S phase transcription. Cell.

[45] Helmrich A., Ballarino M., Tora L. (2011). Collisions between replication and transcription complexes cause common fragile site instability at the longest human genes. Mol. Cell.

[46] Barlow J.H., Faryabi R.B., Callén E., Wong N., Malhowski A., Chen H.T., Gutierrez-Cruz G., Sun H.-W., McKinnon P., Wright G. (2013). Identification of early replicating fragile sites that contribute to genome instability. Cell.

[47] Glover T.W., Berger C., Coyle J., Echo B. (1984). DNA polymerase alpha inhibition by aphidicolin induces gaps and breaks at common fragile sites in human chromosomes. Hum. Genet..

[48] Durkin S.G., Glover T.W. (2007). Chromosome fragile sites. Annu. Rev. Genet..

[49] Elder F.F., Robinson T.J. (1989). Rodent common fragile sites: Are they conserved? Evidence from mouse and rat. Chromosoma.

[50] Raveendranathan M., Chattopadhyay S., Bolon Y.-T., Haworth J., Clarke D.J., Bielinsky A.-K. (2006). Genome-wide replication profiles of S-phase checkpoint mutants reveal fragile sites in yeast. EMBO J..

[51] Helmrich A., Stout-Weider K., Hermann K., Schrock E., Heiden T. (2006). Common fragile sites are conserved features of human and mouse chromosomes and relate to large active genes. Genome Res..

[52] Burrow A.A., Williams L.E., Pierce L.C.T., Wang Y.-H. (2009). Over half of breakpoints in gene pairs involved in cancer-specific recurrent translocations are mapped to human chromosomal fragile sites. BMC Genomics.

[53] Fungtammasan A., Walsh E., Chiaromonte F., Eckert K.A., Makova K.D. (2012). A genome-wide analysis of common fragile sites: What features determine chromosomal instability in the human genome?. Genome Res..

[54] Zhang H., Freudenreich C.H. (2007). An AT-rich sequence in human common fragile site FRA16D causes fork stalling and chromosome breakage in *S. cerevisiae*. Mol. Cell.

[55] Dillon L.W., Pierce L.C.T., Ng M.C.Y., Wang Y.-H. (2013). Role of DNA secondary structures in fragile site breakage along human chromosome 10. Hum. Mol. Genet..

[56] Corbin S., Neilly M.E., Espinosa R., Davis E.M., McKeithan T.W., Le Beau M.M. (2002). Identification of unstable sequences within the common fragile site at 3p14.2: Implications for the mechanism of deletions within fragile histidine triad gene/common fragile site at 3p14.2 in tumors. Cancer Res..

[57] Finnis M., Dayan S., Hobson L., Chenevix-Trench G., Friend K., Ried K., Venter D., Woollatt E., Baker E., Richards R.I. (2005). Common chromosomal fragile site FRA16D mutation in cancer cells. Hum. Mol. Genet..

[58] Debatisse M., Le Tallec B., Letessier A., Dutrillaux B., Brison O. (2012). Common fragile sites: Mechanisms of instability revisited. Trends Genet..

[59] Palumbo E., Matricardi L., Tosoni E., Bensimon A., Russo A. (2010). Replication dynamics at common fragile site FRA6E. Chromosoma.

[60] Letessier A., Millot G.A., Koundrioukoff S., Lachagès A.-M., Vogt N., Hansen R.S., Malfoy B., Brison O., Debatisse M. (2011). Cell-type-specific replication initiation programs set fragility of the FRA3B fragile site. Nature.

[61] Le Tallec B., Dutrillaux B., Lachages A.-M., Millot G.A., Brison O., Debatisse M. (2011). Molecular profiling of common fragile sites in human fibroblasts. Nat. Struct. Mol. Biol..

[62] Cayrou C., Coulombe P., Méchali M. (2010). Programming DNA replication origins and chromosome organization. Chromosome Res..

[63] Dazy S., Gandrillon O., Hyrien O., Prioleau M.-N. (2006). Broadening of DNA replication origin usage during metazoan cell differentiation. EMBO Rep..

[64] Weddington N., Stuy A., Hiratani I., Ryba T., Yokochi T., Gilbert D.M. (2008). ReplicationDomain: A visualization tool and comparative database for genome-wide replication timing data. BMC Bioinform..

[65] Hansen R.S., Thomas S., Sandstrom R., Canfield T.K., Thurman R.E., Weaver M., Dorschner M.O., Gartler S.M., Stamatoyannopoulos J.A. (2010). Sequencing newly replicated DNA reveals widespread plasticity in human replication timing. Proc. Natl. Acad. Sci. USA.

[66] Le Tallec B., Millot G.A., Blin M.E., Brison O., Dutrillaux B., Debatisse M. (2013). Common fragile site profiling in epithelial and erythroid cells reveals that most recurrent cancer deletions lie in fragile sites hosting large genes. Cell Rep..

[67] Le Tallec B., Koundrioukoff S., Wilhelm T., Letessier A., Brison O., Debatisse M. (2014). Updating the mechanisms of common fragile site instability: How to reconcile the different views?. Cell. Mol. Life Sci..

[68] Le Beau M. (1998). Replication of a common fragile site, FRA3B, occurs late in S phase and is delayed further upon induction: Implications for the mechanism of fragile site induction. Hum. Mol. Genet..

[69] El Achkar E., Gerbault-Seureau M., Muleris M., Dutrillaux B., Debatisse M. (2005). Premature condensation induces breaks at the interface of early and late replicating chromosome bands bearing common fragile sites. Proc. Natl. Acad. Sci. USA.

[70] Naim V., Rosselli F. (2009). The FANC pathway and BLM collaborate during mitosis to prevent micro-nucleation and chromosome abnormalities. Nat. Cell Biol..

[71] Bergoglio V., Boyer A.-S., Walsh E., Naim V., Legube G., Lee M.Y., Rey L., Rosselli F., Cazaux C., Eckert K.A. (2013). DNA synthesis by Pol η promotes fragile site stability by preventing under-replicated DNA in mitosis. J. Cell Biol..

[72] Koundrioukoff S., Carignon S., Técher H., Letessier A., Brison O., Debatisse M. (2013). Stepwise activation of the ATR signaling pathway upon increasing replication stress impacts fragile site integrity. PLoS Genet..

[73] Sfeir A., Kosiyatrakul S.T., Hockemeyer D., MacRae S.L., Karlseder J., Schildkraut C.L., de Lange T. (2009). Mammalian telomeres resemble fragile sites and require TRF1 for efficient replication. Cell.

[74] Vannier J.-B., Pavicic-Kaltenbrunner V., Petalcorin M.I.R., Ding H., Boulton S.J. (2012). RTEL1 dismantles T loops and counteracts telomeric G4-DNA to maintain telomere integrity. Cell.

[75] Martínez P., Thanasoula M., Muñoz P., Liao C., Tejera A., McNees C., Flores J.M., Fernández-Capetillo O., Tarsounas M., Blasco M.A. (2009). Increased telomere fragility and fusions resulting from TRF1 deficiency lead to degenerative pathologies and increased cancer in mice. Genes Dev..

[76] Martínez P., Flores J.M., Blasco M. (2012). A 53BP1 deficiency combined with telomere dysfunction activates ATR-dependent DNA damage response. J. Cell Biol..

[77] Westhorpe F.G., Straight A.F. (2013). Functions of the centromere and kinetochore in chromosome segregation. Curr. Opin. Cell Biol..

[78] Willard H.F. (1991). Evolution of alpha satellite. Curr. Opin. Genet. Dev..

[79] Waye J.S., Willard H.F. (1987). Nucleotide sequence heterogeneity of alpha satellite repetitive DNA: A survey of alphoid sequences from different human chromosomes. Nucleic Acids Res..

[80] Vissel B., Choo K.H. (1987). Human alpha satellite DNA—Consensus sequence and conserved regions. Nucleic Acids Res..

[81] Greenfeder S.A., Newlon C.S. (1992). Replication forks pause at yeast centromeres. Mol. Cell. Biol..

[82] Simi S., Simili M., Bonatti S., Campagna M., Abbondandolo A. (1998). Fragile sites at the centromere of Chinese hamster chromosomes: A possible mechanism of chromosome loss. Mutat. Res..

[83] Sundin O., Varshavsky A. (1980). Terminal stages of SV40 DNA replication proceed via multiply intertwined catenated dimers. Cell.

[84] McFarlane R.J., Humphrey T.C. (2010). A role for recombination in centromere function. Trends Genet..

[85] Chen Y.-H., Jones M.J.K., Yin Y., Crist S.B., Colnaghi L., Sims R.J., Rothenberg E., Jallepalli P.V., Huang T.T. (2015). ATR-mediated phosphorylation of FANCI regulates dormant origin firing in response to replication stress. Mol. Cell.

[86] McIntosh D., Blow J.J. (2012). Dormant origins, the licensing checkpoint, and the response to replicative stresses. Cold Spring Harb. Perspect. Biol..

[87] Haber J.E. (2014). Genome Stability: DNA Repair and Recombinaition.

[88] Saintigny Y. (2001). Characterization of homologous recombination induced by replication inhibition in mammalian cells. EMBO J..

[89] Grabarz A., Guirouilh-Barbat J., Barascu A., Pennarun G., Genet D., Rass E., Germann S.M., Bertrand P., Hickson I.D., Lopez B.S. (2013). A role for BLM in double-strand break repair pathway choice: Prevention of CtIP/Mre11-mediated alternative nonhomologous end-joining. Cell Rep..

[90] Guirouilh-Barbat J., Huck S., Bertrand P., Pirzio L., Desmaze C., Sabatier L., Lopez B.S. (2004). Impact of the KU80 pathway on NHEJ-induced genome rearrangements in mammalian cells. Mol. Cell.

[91] Di Virgilio M., Callen E., Yamane A., Zhang W., Jankovic M., Gitlin A.D., Feldhahn N., Resch W., Oliveira T.Y., Chait B.T. (2013). Rif1 prevents resection of DNA breaks and promotes immunoglobulin class switching. Science.

[92] Zimmermann M., Lottersberger F., Buonomo S.B., Sfeir A., de Lange T. (2013). 53BP1 regulates DSB repair using Rif1 to control 5′ end resection. Science.

[93] Bunting S.F., Callén E., Wong N., Chen H.T., Polato F., Gunn A., Bothmer A., Feldhahn N., Fernandez-Capetillo O., Cao L. (2010). 53BP1 inhibits homologous recombination in brca1-deficient cells by blocking resection of DNA breaks. Cell.

[94] Reina-San-Martin B., Chen J., Nussenzweig A.A., Nussenzweig M.C. (2007). Enhanced intra-switch region recombination during immunoglobulin class switch recombination in 53BP1−/− B cells. Eur. J. Immunol..

[95] Huertas P., Jackson S.P. (2009). Human CtIP mediates cell cycle control of DNA end resection and double strand break repair. J. Biol. Chem..

[96] Yu X., Chen J. (2004). DNA damage-induced cell cycle checkpoint control requires CtIP, a phosphorylation-dependent binding partner of BRCA1 C-terminal domains. Mol. Cell. Biol..

[97] Sartori A.A., Lukas C., Coates J., Mistrik M., Fu S., Bartek J., Baer R., Lukas J., Jackson S.P. (2007). Human CtIP promotes DNA end resection. Nature.

[98] Langerak P., Mejia-Ramirez E., Limbo O., Russell P. (2011). Release of Ku and MRN from DNA ends by Mre11 nuclease activity and Ctp1 is required for homologous recombination repair of double-strand breaks. PLoS Genet..

[99] Rass E., Grabarz A., Plo I., Gautier J., Bertrand P., Lopez B.S. (2009). Role of Mre11 in chromosomal nonhomologous end joining in mammalian cells. Nat. Struct. Mol. Biol..

[100] Escribano-Díaz C., Orthwein A., Fradet-Turcotte A., Xing M., Young J.T.F., Tkáč J., Cook M.A., Rosebrock A.P., Munro M., Canny M.D. (2013). A cell cycle-dependent regulatory circuit composed of 53BP1-RIF1 and BRCA1-CtIP controls DNA repair pathway choice. Mol. Cell.

[101] Feng L., Fong K.W., Wang J., Wang W., Chen J. (2013). RIF1 counteracts BRCA1-mediated end resection during DNA repair. J. Biol. Chem..

[102] Chapman J.R., Sossick A.J., Boulton S.J., Jackson S.P. (2012). BRCA1-associated exclusion of 53BP1 from DNA damage sites underlies temporal control of DNA repair. J. Cell Sci..

[103] Tomimatsu N., Mukherjee B., Hardebeck M.C., Ilcheva M., Camacho C.V., Harris J.L., Porteus M., Llorente B. (2014). Phosphorylation of EXO1 by CDKs 1 and 2 regulates DNA end resection and repair pathway choice Nozomi. Nat. Commun..

[104] Xu G., Chapman J.R., Brandsma I., Yuan J., Mistrik M., Bouwman P., Bartkova J., Gogola E., Warmerdam D., Barazas M. (2015). REV7 counteracts DNA double-strand break resection and affects PARP inhibition. Nature.

[105] Boersma V., Moatti N., Segura-Bayona S., Peuscher M.H., van der Torre J., Wevers B.A., Orthwein A., Durocher D., Jacobs J.J.L. (2015). MAD2L2 controls DNA repair at telomeres and DNA breaks by inhibiting 5′ end resection. Nature.

[106] Potts P.R., Porteus M.H., Yu H. (2006). Human SMC5/6 complex promotes sister chromatid homologous recombination by recruiting the SMC1/3 cohesin complex to double-strand breaks. EMBO J..

[107] Xu H., Balakrishnan K., Malaterre J., Beasley M., Yan Y., Essers J., Appeldoorn E., Tomaszewski J.M., Thomaszewski J.M., Vazquez M. (2010). Rad21-cohesin haploinsufficiency impedes DNA repair and enhances gastrointestinal radiosensitivity in mice. PLoS ONE.

[108] De Piccoli G., Cortes-Ledesma F., Ira G., Torres-Rosell J., Uhle S., Farmer S., Hwang J.-Y., Machin F., Ceschia A., McAleenan A. (2006). Smc5-Smc6 mediate DNA double-strand-break repair by promoting sister-chromatid recombination. Nat. Cell Biol..

[109] Nickoloff J.A. (1992). Transcription enhances intrachromosomal homologous recombination in mammalian cells. Mol. Cell. Biol..

[110] Aymard F., Bugler B., Schmidt C.K., Guillou E., Caron P., Briois S., Iacovoni J.S., Daburon V., Miller K.M., Jackson S.P. (2014). Transcriptionally active chromatin recruits homologous recombination at DNA double-strand breaks. Nat. Struct. Mol. Biol..

[111] Lopes M., Cotta-Ramusino C., Pellicioli A., Liberi G., Plevani P., Muzi-Falconi M., Newlon C.S., Foiani M. (2001). The DNA replication checkpoint response stabilizes stalled replication forks. Nature.

[112] Cobb J.A., Bjergbaek L., Shimada K., Frei C., Gasser S.M. (2003). DNA polymerase stabilization at stalled replication forks requires Mec1 and the RecQ helicase Sgs. EMBO J..

[113] Lucca C., Vanoli F., Cotta-Ramusino C., Pellicioli A., Liberi G., Haber J., Foiani M. (2004). Checkpoint-mediated control of replisome-fork association and signalling in response to replication pausing. Oncogene.

[114] Hashimoto Y., Ray Chaudhuri A., Lopes M., Costanzo V. (2010). Rad51 protects nascent DNA from Mre11-dependent degradation and promotes continuous DNA synthesis. Nat. Struct. Mol. Biol..

[115] Hashimoto Y., Puddu F., Costanzo V. (2011). RAD51- and MRE11-dependent reassembly of uncoupled CMG helicase complex at collapsed replication forks. Nat. Struct. Mol. Biol..

[116] Schlacher K., Christ N., Siaud N., Egashira A., Wu H., Jasin M. (2011). Double-strand break repair-independent role for BRCA2 in blocking stalled replication fork degradation by MRE11. Cell.

[117] Schlacher K., Wu H., Jasin M. (2012). A distinct replication fork protection pathway connects fanconi anemia tumor suppressors to RAD51-BRCA1/2. Cancer Cell.

[118] Ying S., Hamdy F.C., Helleday T. (2012). Mre11-dependent degradation of stalled DNA replication forks is prevented by BRCA2 and PARP1. Cancer Res..

[119] Pefani D.-E., Latusek R., Pires I., Grawenda A.M., Yee K.S., Hamilton G., van der Weyden L., Esashi F., Hammond E.M., O’Neill E. (2014). RASSF1A-LATS1 signalling stabilizes replication forks by restricting CDK2-mediated phosphorylation of BRCA2. Nat. Cell Biol..

[120] Higgins N.P., Kato K., Strauss B. (1976). A model for replication repair in mammalian cells. J. Mol. Biol..

[121] Seigneur M., Bidnenko V., Ehrlich S.D., Michel B. (1998). RuvAB acts at arrested replication forks. Cell.

[122] Sogo J.M., Lopes M., Foiani M. (2002). Fork reversal and ssDNA accumulation at stalled replication forks owing to checkpoint defects. Science.

[123] Ray Chaudhuri A., Hashimoto Y., Herrador R., Neelsen K.J., Fachinetti D., Bermejo R., Cocito A., Costanzo V., Lopes M. (2012). Topoisomerase I poisoning results in PARP-mediated replication fork reversal. Nat. Struct. Mol. Biol..

[124] Bansbach C.E., Bétous R., Lovejoy C.A., Glick G.G., Cortez D. (2009). The annealing helicase SMARCAL1 maintains genome integrity at stalled replication forks. Genes Dev..

[125] Zellweger R., Dalcher D., Mutreja K., Berti M., Schmid J.A., Herrador R., Vindigni A., Lopes M. (2015). Rad51-mediated replication fork reversal is a global response to genotoxic treatments in human cells. J. Cell Biol..

[126] Ragland R.L., Patel S., Rivard R.S., Smith K., Peters A.A., Bielinsky A.-K., Brown E.J. (2013). RNF4 and PLK1 are required for replication fork collapse in ATR-deficient cells. Genes Dev..

[127] Toledo L.I., Murga M., Fernandez-Capetillo O. (2011). Targeting ATR and Chk1 kinases for cancer treatment: A new model for new (and old) drugs. Mol. Oncol..

[128] Nam E.A., Cortez D. (2011). ATR signalling: More than meeting at the fork. Biochem. J..

[129] Labib K., de Piccoli G. (2011). Surviving chromosome replication: The many roles of the S-phase checkpoint pathway. Philos. Trans. R. Soc. Lond. B Biol. Sci..

[130] Mizuno K., Lambert S., Baldacci G., Murray J.M., Carr A.M. (2009). Nearby inverted repeats fuse to generate acentric and dicentric palindromic chromosomes by a replication template exchange mechanism. Genes Dev..

[131] Lambert S., Mizuno K., Blaisonneau J., Martineau S., Chanet R., Fréon K., Murray J.M., Carr A.M., Baldacci G. (2010). Homologous recombination restarts blocked replication forks at the expense of genome rearrangements by template exchange. Mol. Cell.

[132] Neelsen K.J., Zanini I.M.Y., Herrador R., Lopes M. (2013). Oncogenes induce genotoxic stress by mitotic processing of unusual replication intermediates. J. Cell Biol..

[133] Matos J., Blanco M.G., Maslen S., Skehel J.M., West S.C. (2011). Regulatory control of the resolution of DNA recombination intermediates during meiosis and mitosis. Cell.

[134] Pageau G.J., Lawrence J.B. (2006). BRCA1 foci in normal S-phase nuclei are linked to interphase centromeres and replication of pericentric heterochromatin. J. Cell Biol..

[135] Buonomo S.B.C., Wu Y., Ferguson D., de Lange T. (2009). Mammalian Rif1 contributes to replication stress survival and homology-directed repair. J. Cell Biol..

[136] Nakamura K., Okamoto A., Katou Y., Yadani C., Shitanda T., Kaweeteerawat C., Takahashi T.S., Itoh T., Shirahige K., Masukata H. (2008). Rad51 suppresses gross chromosomal rearrangement at centromere in Schizosaccharomyces pombe. EMBO J..

[137] Jaco I., Canela A., Vera E., Blasco M.A. (2008). Centromere mitotic recombination in mammalian cells. J. Cell Biol..

[138] Daboussi F., Courbet S., Benhamou S., Kannouche P., Zdzienicka M.Z., Debatisse M., Lopez B.S. (2008). A homologous recombination defect affects replication-fork progression in mammalian cells. J. Cell Sci..

[139] Guirouilh-Barbat J., Lambert S., Bertrand P., Lopez B.S. (2014). Is homologous recombination really an error-free process?. Front. Genet..

[140] Crasta K., Ganem N.J., Dagher R., Lantermann A.B., Ivanova E.V., Pan Y., Nezi L., Protopopov A., Chowdhury D., Pellman D. (2012). DNA breaks and chromosome pulverization from errors in mitosis. Nature.

[141] Jones M.J.K., Jallepalli P.V. (2012). Chromothripsis: Chromosomes in crisis. Dev. Cell.

[142] Forment J.V., Kaidi A., Jackson S.P. (2012). Chromothripsis and cancer: Causes and consequences of chromosome shattering. Nat. Rev. Cancer.

[143] Liu P., Erez A., Nagamani S.C.S., Dhar S.U., Ko K.E., Dharmadhikari A.V., Cooper M.L., Zhang F., Withers M.A., Bacino C.A. (2012). Chromosome catastrophes involve replication mechanisms generating complex genomic rearrangements. Cell.

[144] Lee J.A., Carvalho C.M.B., Lupski J.R. (2007). A DNA replication mechanism for generating nonrecurrent rearrangements associated with genomic disorders. Cell.

[145] Zhang F., Khajavi M., Connolly A.M., Towne C.F., Batish S.D., Lupski J.R. (2009). The DNA replication FoSTeS/MMBIR mechanism can generate genomic, genic and exonic complex rearrangements in humans. Nat. Genet..

[146] Roseaulin L., Yamada Y., Tsutsui Y., Russell P., Iwasaki H., Arcangioli B. (2008). Mus81 is essential for sister chromatid recombination at broken replication forks. EMBO J..

[147] Hanada K., Budzowska M., Davies S.L., van Drunen E., Onizawa H., Beverloo H.B., Maas A., Essers J., Hickson I.D., Kanaar R. (2007). The structure-specific endonuclease Mus81 contributes to replication restart by generating double-strand DNA breaks. Nat. Struct. Mol. Biol..

[148] Bétermier M., Bertrand P., Lopez B.S. (2014). Is non-homologous end-joining really an inherently error-prone process?. PLoS Genet..

[149] Bothmer A., Robbiani D.F., di Virgilio M., Bunting S.F., Klein I.A., Feldhahn N., Barlow J., Chen H.-T.T., Bosque D., Callen E. (2011). Regulation of DNA end joining, resection, and immunoglobulin class switch recombination by 53BP1. Mol. Cell.

[150] Bunting S.F., Nussenzweig A. (2013). End-joining, translocations and cancer. Nat. Rev. Cancer.

[151] Deriano L., Roth D.B. (2013). Modernizing the nonhomologous end-joining repertoire: Alternative and classical NHEJ share the stage. Annu. Rev. Genet..

[152] Chen X., Niu H., Chung W.-H., Zhu Z., Papusha A., Shim E.Y., Lee S.E., Sung P., Ira G. (2011). Cell cycle regulation of DNA double-strand break end resection by Cdk1-dependent Dna2 phosphorylation. Nat. Struct. Mol. Biol..

[153] Yousefzadeh M.J., Wyatt D.W., Takata K.-I., Mu Y., Hensley S.C., Tomida J., Bylund G.O., Doublié S., Johansson E., Ramsden D.A. (2014). Mechanism of suppression of chromosomal instability by DNA polymerase POLQ. PLoS Genet..

[154] Mateos-gomez P.A., Gong F., Nair N., Miller K.M., Lazzerini-denchi E., Sfeir A. (2015). Mammalian polymerase θ promotes alternative NHEJ and suppresses recombination. Nature.

[155] Sturzenegger A., Burdova K., Kanagaraj R., Levikova M., Pinto C., Cejka P., Janscak P. (2014). DNA2 cooperates with the WRN and BLM RecQ helicases to mediate long-range DNA-end resection in human cells. J. Biol. Chem..

[156] Couëdel C., Mills K.D., Barchi M., Shen L., Olshen A., Johnson R.D., Nussenzweig A., Essers J., Kanaar R., Li G.C. (2004). Collaboration of homologous recombination and nonhomologous end-joining factors for the survival and integrity of mice and cells. Genes Dev..

[157] Mills K.D., Ferguson D.O., Essers J., Eckersdorff M., Kanaar R., Alt F.W. (2004). Rad54 and DNA Ligase IV cooperate to maintain mammalian chromatid stability. Genes Dev..

[158] Adamo A., Collis S.J., Adelman C.A., Silva N., Horejsi Z., Ward J.D., Martinez-Perez E., Boulton S.J., La Volpe A. (2010). Preventing nonhomologous end joining suppresses DNA repair defects of fanconi anemia. Mol. Cell.

[159] Pace P., Mosedale G., Hodskinson M.R., Rosado I.V., Sivasubramaniam M., Patel K.J. (2010). Ku70 corrupts DNA repair in the absence of the Fanconi anemia pathway. Science.

[160] Smogorzewska A., Karlseder J., Holtgreve-Grez H., Jauch A., de Lange T. (2002). DNA ligase IV-dependent NHEJ of deprotected mammalian telomeres in G1 and G2. Curr. Biol..

[161] Celli G.B., de Lange T. (2005). DNA processing is not required for ATM-mediated telomere damage response after TRF2 deletion. Nat. Cell Biol..

[162] Celli G.B., Denchi E.L., de Lange T. (2006). Ku70 stimulates fusion of dysfunctional telomeres yet protects chromosome ends from homologous recombination. Nat. Cell Biol..

[163] Aten J.A., Stap J., Krawczyk P.M., van Oven C.H., Hoebe R.A., Essers J., Kanaar R. (2004). Dynamics of DNA double-strand breaks revealed by clustering of damaged chromosome domains. Science.

[164] Kruhlak M.J., Celeste A., Dellaire G., Fernandez-Capetillo O., Müller W.G., McNally J.G., Bazett-Jones D.P., Nussenzweig A. (2006). Changes in chromatin structure and mobility in living cells at sites of DNA double-strand breaks. J. Cell Biol..

[165] Krawczyk P.M., Borovski T., Stap J., Cijsouw T., ten Cate R., Medema J.P., Kanaar R., Franken N.A.P., Aten J.A. (2012). Chromatin mobility is increased at sites of DNA double-strand breaks. J. Cell Sci..

[166] Soutoglou E., Dorn J.F., Sengupta K., Jasin M., Nussenzweig A., Ried T., Danuser G., Misteli T. (2007). Positional stability of single double-strand breaks in mammalian cells. Nat. Cell Biol..

[167] Roukos V., Voss T.C., Schmidt C.K., Lee S., Wangsa D., Misteli T. (2013). Spatial dynamics of chromosome translocations in living cells. Science.

[168] Girst S., Hable V., Drexler G.A., Greubel C., Siebenwirth C., Haum M., Friedl A.A., Dollinger G. (2013). Subdiffusion supports joining of correct ends during repair of DNA double-strand breaks. Sci. Rep..

[169] Becker A., Durante M., Taucher-Scholz G., Jakob B. (2014). ATM alters the otherwise robust chromatin mobility at sites of DNA double-strand breaks (DSBs) in human cells. PLoS ONE.

[170] Krenning L., Feringa F.M., Shaltiel I.A., vanden Berg J., Medema R.H. (2014). Transient activation of p53 in G2 phase is sufficient to induce senescence. Mol. Cell.

[171] Müllers E., Cascales H.S., Jaiswal H., Saurin A.T., Lindqvist A. (2014). Translocation of Cyclin B1 marks the restriction point for terminal cell cycle exit in G2 phase. Cell Cycle.

[172] Chan K.L., Palmai-Pallag T., Ying S., Hickson I.D. (2009). Replication stress induces sister-chromatid bridging at fragile site loci in mitosis. Nat. Cell Biol..

[173] Baumann C., Körner R., Hofmann K., Nigg E.A. (2007). PICH, a centromere-associated SNF2 family atpase, is regulated by plk1 and required for the spindle checkpoint. Cell.

[174] Vinciguerra P., Godinho S.A., Parmar K., Pellman D., D’Andrea A.D. (2010). Cytokinesis failure occurs in Fanconi anemia pathway-deficient murine and human bone marrow hematopoietic cells. J. Clin. Investig..

[175] Naim V., Wilhelm T., Debatisse M., Rosselli F. (2013). ERCC1 and MUS81-EME1 promote sister chromatid separation by processing late replication intermediates at common fragile sites during mitosis. Nat. Cell Biol..

[176] Liu Y., Nielsen C.F., Yao Q., Hickson I.D. (2014). The origins and processing of ultra fine anaphase DNA bridges. Curr. Opin. Genet. Dev..

[177] Guervilly J.-H., Takedachi A., Naim V., Scaglione S., Chawhan C., Lovera Y., Despras E., Kuraoka I., Kannouche P., Rosselli F. (2015). The SLX4 Complex Is a SUMO E3 Ligase that Impacts on Replication Stress Outcome and Genome Stability. Mol. Cell.

[178] Chu W.K., Hickson I.D. (2009). RecQ helicases: Multifunctional genome caretakers. Nat. Rev. Cancer.

[179] Barefield C., Karlseder J. (2012). The BLM helicase contributes to telomere maintenance through processing of late-replicating intermediate structures. Nucleic Acids Res..

[180] Lahkim Bennani-Belhaj K., Rouzeau S., Buhagiar-Labarchède G., Chabosseau P., Onclercq-Delic R., Bayart E., Cordelières F., Couturier J., Amor-Guéret M. (2010). The Bloom syndrome protein limits the lethality associated with RAD51 deficiency. Mol. Cancer Res..

[181] Laulier C., Cheng A., Stark J.M. (2011). The relative efficiency of homology-directed repair has distinct effects on proper anaphase chromosome separation. Nucleic Acids Res..

[182] Rodrigue A., Coulombe Y., Jacquet K., Gagné J.-P., Roques C., Gobeil S., Poirier G., Masson J.-Y. (2013). The RAD51 paralogs ensure cellular protection against mitotic defects and aneuploidy. J. Cell Sci..

[183] Tahara H., Shin-Ya K., Seimiya H., Yamada H., Tsuruo T., Ide T. (2006). G-Quadruplex stabilization by telomestatin induces TRF2 protein dissociation from telomeres and anaphase bridge formation accompanied by loss of the 3′ telomeric overhang in cancer cells. Oncogene.

[184] Van Steensel B., Smogorzewska A., de Lange T. (1998). TRF2 protects human telomeres from end-to-end fusions. Cell.

[185] Skoufias D.A., Andreassen P.R., Lacroix F.B., Wilson L., Margolis R.L. (2001). Mammalian mad2 and bub1/bubR1 recognize distinct spindle-attachment and kinetochore-tension checkpoints. Proc. Natl. Acad. Sci. USA.

[186] Cimini D., Tanzarella C., Degrassi F. (1999). Differences in malsegregation rates obtained by scoring ana-telophases or binucleate cells. Mutagenesis.

[187] Griffin C.S., Simpson P.J., Wilson C.R., Thacker J. (2000). Mammalian recombination-repair genes XRCC2 and XRCC3 promote correct chromosome segregation. Nat. Cell Biol..

[188] Kraakman-van der Zwet M., Overkamp W.J.I., van Lange R.E.E., Essers J., van Duijn-Goedhart A., Wiggers I., Swaminathan S., van Buul P.P.W., Errami A., Tan R.T.L. (2002). Brca2 (XRCC11) deficiency results in radioresistant DNA synthesis and a higher frequency of spontaneous deletions. Mol. Cell. Biol..

[189] Bertrand P., Lambert S., Joubert C., Lopez B.S. (2003). Overexpression of mammalian Rad51 does not stimulate tumorigenesis while a dominant-negative Rad51 affects centrosome fragmentation, ploidy and stimulates tumorigenesis, in p53-defective CHO cells. Oncogene.

[190] Daboussi F., Thacker J., Lopez B.S. (2005). Genetic interactions between RAD51 and its paralogues for centrosome fragmentation and ploidy control, independently of the sensitivity to genotoxic stresses. Oncogene.

[191] Rattner J.B., Phillips S.G. (1973). Independence of centriole formation and DNA synthesis. J. Cell Biol..

[192] Balczon R., Bao L., Zimmer W.E., Brown K., Zinkowski R.P., Brinkley B.R. (1995). Dissociation of centrosome replication events from cycles of DNA synthesis and mitotic division in hydroxyurea-arrested Chinese hamster ovary cells. J. Cell Biol..

[193] Wong C., Stearns T. (2003). Centrosome number is controlled by a centrosome-intrinsic block to reduplication. Nat. Cell Biol..

[194] Katsura M., Tsuruga T., Date O., Yoshihara T., Ishida M., Tomoda Y., Okajima M., Takaku M., Kurumizaka H., Kinomura A. (2009). The ATR-Chk1 pathway plays a role in the generation of centrosome aberrations induced by Rad51C dysfunction. Nucleic Acids Res..

[195] Goepfert T.M., Adigun Y.E., Zhong L., Gay J., Medina D., Brinkley W.R. (2002). Centrosome amplification and overexpression of aurora a are early events in rat mammary carcinogenesis. Cancer Res..

[196] Pihan G.A., Wallace J., Zhou Y., Doxsey S.J. (2003). Centrosome abnormalities and chromosome instability occur together in pre-invasive carcinomas. Cancer Res..

[197] Liontos M., Koutsami M., Sideridou M., Evangelou K., Kletsas D., Levy B., Kotsinas A., Nahum O., Zoumpourlis V., Kouloukoussa M. (2007). Deregulated overexpression of hCdt1 and hCdc6 promotes malignant behavior. Cancer Res..

[198] Hontz A.E., Li S.A., Lingle W.L., Negron V., Bruzek A., Salisbury J.L., Li J.J. (2007). Aurora A and B overexpression and centrosome amplification in early estrogen-induced tumor foci in the Syrian hamster kidney: Implications for chromosomal instability, aneuploidy, and neoplasia. Cancer Res..

[199] Duensing A., Chin A., Wang L., Kuan S.-F., Duensing S. (2008). Analysis of centrosome overduplication in correlation to cell division errors in high-risk human papillomavirus (HPV)-associated anal neoplasms. Virology.

[200] Howell W.H. (1890). The life-history of the formed elements of the blood, especially the red blood corpuscles. J. Morphol..

[201] Jolly M. (1907). Recherches sur la formation des globules rouges des mammiferes. Arch. D’anatomie Microsc..

[202] Dawson D.W., Bury H.P. (1961). The significance of Howell-Jolly bodies and giant metamyelocytes in marrow smears. J. Clin. Pathol..

[203] Thompson S.L., Compton D.A. (2011). Chromosome missegregation in human cells arises through specific types of kinetochore-microtubule attachment errors. Proc. Natl. Acad. Sci. USA.

[204] Gisselsson D., Björk J., Höglund M., Mertens F., dal Cin P., Akerman M., Mandahl N. (2001). Abnormal nuclear shape in solid tumors reflects mitotic instability. Am. J. Pathol..

[205] Terradas M., Martín M., Tusell L., Genescà A. (2010). Genetic activities in micronuclei: Is the DNA entrapped in micronuclei lost for the cell?. Mutat. Res. Rev. Mutat. Res..

[206] Savage J.R. (1988). A comment on the quantitative relationship between micronuclei and chromosomal aberrations. Mutat. Res..

[207] Fenech M. (2007). Cytokinesis-block micronucleus cytome assay. Nat. Protoc..

[208] Fenech M., Kirsch-Volders M., Natarajan A.T., Surralles J., Crott J.W., Parry J., Norppa H., Eastmond D.A., Tucker J.D., Thomas P. (2011). Molecular mechanisms of micronucleus, nucleoplasmic bridge and nuclear bud formation in mammalian and human cells. Mutagenesis.

[209] Xu B., Sun Z., Liu Z., Guo H., Liu Q., Jiang H., Zou Y., Gong Y., Tischfield J.A., Shao C. (2011). Replication stress induces micronuclei comprising of aggregated DNA double-strand breaks. PLoS ONE.

[210] Li M., Fang X., Baker D.J., Guo L., Gao X., Wei Z., Han S., van Deursen J.M., Zhang P. (2010). The ATM-p53 pathway suppresses aneuploidy-induced tumorigenesis. Proc. Natl. Acad. Sci. USA.

[211] Thompson S.L., Compton D.A. (2010). Proliferation of aneuploid human cells is limited by a p53-dependent mechanism. J. Cell Biol..

[212] Harrigan J.A., Belotserkovskaya R., Coates J., Dimitrova D.S., Polo S.E., Bradshaw C.R., Fraser P., Jackson S.P. (2011). Replication stress induces 53BP1-containing OPT domains in G1 cells. J. Cell Biol..

[213] Lukas C., Savic V., Bekker-Jensen S., Doil C., Neumann B., Pedersen R.S., Grøfte M., Chan K.L., Hickson I.D., Bartek J. (2011). 53BP1 nuclear bodies form around DNA lesions generated by mitotic transmission of chromosomes under replication stress. Nat. Cell Biol..

[214] Benada J., Burdová K., Lidak T., von Morgen P., Macurek L., Benada J., Burdov K. (2015). Polo-like kinase 1 inhibits DNA damage response during mitosis. Cell Cycle.

[215] Orthwein A., Fradet-Turcotte A., Noordermeer S.M., Canny M.D., Brun C.M., Strecker J., Escribano-Diaz C., Durocher D. (2014). Mitosis inhibits DNA double-strand break repair to guard against telomere fusions. Science.

[216] Lee D.-H., Acharya S.S., Kwon M., Drane P., Guan Y., Adelmant G., Kalev P., Shah J., Pellman D., Marto J.A. (2014). Dephosphorylation enables the recruitment of 53BP1 to double-strand DNA breaks. Mol. Cell.

[217] Boveri T., Gustav Fischer J. (1914). Zur frage der entstehung maligner tumoren. Science.

[218] Boveri T. (2008). Concerning the origin of malignant tumours by Theodor Boveri. Translated and annotated by Henry Harris. J. Cell Sci..

